# Mo doping-enhanced dye absorption of Bi_2_Se_3_ nanoflowers

**DOI:** 10.1186/1556-276X-8-451

**Published:** 2013-10-30

**Authors:** Mianzeng Zhong, Xiuqing Meng, Fengmin Wu, Jingbo Li, Yunzhang Fang

**Affiliations:** 1 Research Center for Light Emitting Diodes (LED), Zhejiang Normal University, Jinhua, Zhejiang Province 321004, China

**Keywords:** Solvothermal, Bi_2−*x*_Mo_*x*_Se_3_, Nanoflowers, Nanoplates, Higher adsorption capacity

## Abstract

A simple solvothermal approach is explored to prepare Bi_2−*x*_Mo_*x*_Se_3_ nanostructures by employing *N*,*N*-dimethylformamide (DMF) as the solvent. Mo plays an important role in the assembly of the Bi_2−*x*_Mo_*x*_Se_3_ nanostructures from nanoplates to nanoflowers. Structural and morphological studies indicate that the resulting products are large specific surface area single-crystalline Bi_2−*x*_Mo_*x*_Se_3_ nanoflowers self-assembled from thin nanoplates during the reaction process. The absorption properties of the as-prepared samples are investigated with Rhodamine B (RhB) as dye, and it is found that the Bi_1.85_Mo_0.15_Se_3_ nanoflowers show an optimal adsorption capacity, implying that Mo doping not only changes the morphologies of the nanostructures but also enhances their absorption behaviors.

## Background

Water pollution has now become an urgent problem owing to the rapidly growing global industrial process [1,2]. Public health and social economies are threatened by various organic dye pollutants from textile industries [3]. A variety of methods have been introduced to remove dyes from wastewaters, such as membrane filtration [4], flotation [5,6], solvent extraction [7], chemical oxidation [8,9], adsorption [10,11], and photocatalytic degradation [12,13]. Among these methods, adsorption has been proved to be an effective way for wastewater treatment in terms of simplicity of design, user-friendly control, and insensitivity to toxic substances. Dye removal from industrial wastewaters by adsorption techniques has been widely concerned and researched in recent years [10-15]. Activated carbon is considered one of the best adsorbents for the removal of organic contaminants, but activated carbon is too expensive to use widely in practical applications [16]. Therefore, the development of low-cost, high-efficiency, renewable, and eco-friendly materials as absorbent for the removal of dyes has attracted more and more interests. Recently, many kinds of materials such as SnS_2_ nanosheets [15], WO_3_ nanorods [17], Cu_2_O nanocrystals [18,19], and other highly adsorbent materials have been investigated.

Bismuth selenide (Bi_2_Se_3_) nanostructures have been extensively studied due to their unique properties and promising applications in the fields of optical recording systems, laser materials, optical filters, sensors, solar cells, strain gauges, electromechanical and thermoelectric devices, and topological insulators [20-23]. During the past few years, the preparation and application of doped Bi_2_Se_3_ have been extensively investigated [24-27]. In addition, due to the high surface state and unique optical or electrical properties [28], Bi_2_Se_3_ can also be applied in the fields of visible-light photocatalytic degradation [27,29]. For example, Bi_2_Se_3_-TiO_2_ complex nanobelts [30] and S-doped BiSe [31] show excellent visible-light photocatalytic degradation performance. However, to our knowledge, there is no report on the absorption properties of Bi_2_Se_3_ nanostructures, especially the systematic study of the Mo doping-enhanced absorption behavior of Bi_2_Se_3_ nanostructures.

In this work, we synthesized self-assembled Mo-doped Bi_2_Se_3_ nanoflowers by a simple solvothermal route. We find that the absorption behavior of Bi_2−*x*_Mo_*x*_Se_3_ on Rhodamine B (RhB) varies as a function of Mo content and reaches its highest absorption capacity with 15% Mo doping.

## Methods

### Preparation of Bi_2−*x*_Mo_*x*_Se_3_

All of the chemical reagents used in this experiment are of analytical grade and used without further purification. Bi_2−*x*_Mo_*x*_Se_3_ (*x* = 0, 0.01, 0.03, 0.05, 0.10, and 0.15) is obtained by a simple solvothermal method. In a typical Bi_2−*x*_Mo_*x*_Se_3_ (*x* = 0.15) synthesis, 0.85 mmol of Bi(NO_3_)_3_·5H_2_O and 0.15 mmol of (NH_4_)_6_Mo_7_O_24_·4H_2_O are added to 18 ml of *N*,*N*-dimethylformamide under vigorous stirring to form a homogeneous solution. Then additional ammonia is added to the above solution to adjust the pH value to 9 to 10 under continuous stirring. After that, Se powder and Na_2_SO_3_ are added to the above solution under magnetic stirring. The final solution is transferred into a Teflon-lined autoclave (25-ml capacity), kept at 160°C for 20 h, and cooled to room temperature under ambient conditions. The products are finally washed several times with ethanol and distilled water, followed by drying at 80°C for 12 h under vacuum. For comparison, we also synthesized Bi_2−*x*_Mo_*x*_Se_3_ samples with different Mo contents (*x* = 0, 0.01, 0.03, 0.05, 0.10, and 0.15), which are labeled as samples A, B, C, D, E, and F, respectively.

### Dye adsorption experiments

The adsorption activities of the as-prepared products are investigated using RhB as dyes. In each experiment, 0.08 g of adsorbent was added to 50 ml of a 10-mg/l RhB solution. Under constant stirring in the dark, about 6 ml of the mixture solution is taken out at intervals and centrifuged to separate solid particles for analysis. After centrifugation, the adsorption behavior is investigated.

### Sample characterization

The phase composition and crystallographic structure of the as-prepared samples are examined by X-ray diffraction (XRD) technique with Cu Kα irradiation. The sizes and morphologies of the products are investigated using a field emission scanning electron microscope (FESEM; S-4800, Hitachi, Minato-ku, Tokyo, Japan). The dye adsorption behavior is measured with a UV-visible (UV–vis) spectrum (Lambda 900, PerkinElmer Instruments, Branford, CT, USA).

## Results and discussion

### Structure and morphology

**Figure 1:**
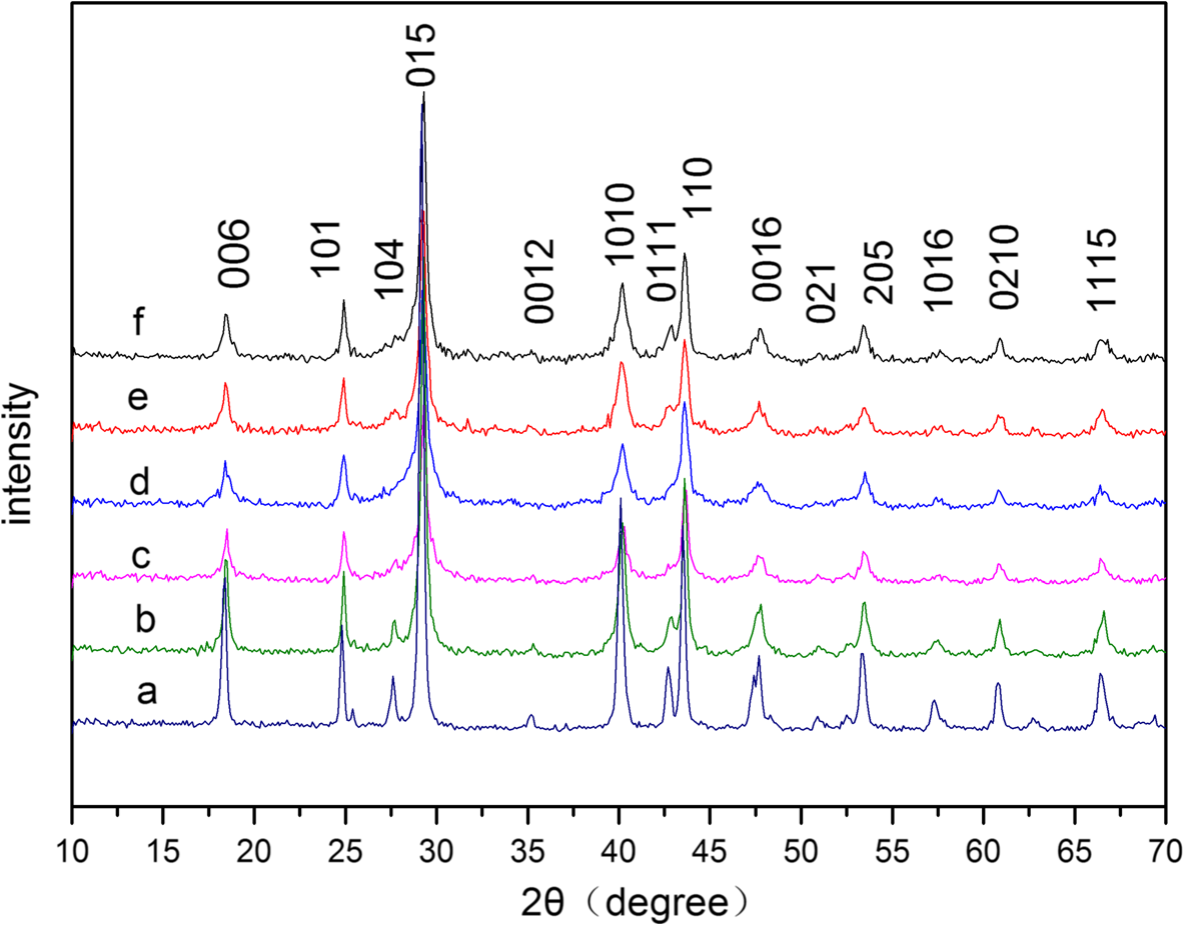
XRD patterns of samples (a) A, (b) B, (c) C, (d) D, (e) E, and (f) F.

The as-prepared samples are examined by XRD techniques, and the XRD patterns of samples A to F are shown in Figure 1. All the peaks in the patterns can be indexed according to the power diffraction card of hexagonal Bi_2_Se_3_ (no. 33-0214), and no impurity phase related to the Mo complex could be found. The diffraction peaks shift to higher angles with the increase of Mo^6+^ content from samples A to F, indicating that Mo^6+^ has been incorporated in the Bi_2_Se_3_ lattice, and the lattice parameter gets smaller with the increase of Mo^6+^. This is understandable considering the fact that the ionic radius of Mo^6+^ (0.065 nm) [24] is smaller than that of Bi^3+^ (0.103 nm) [25].

**Figure 2:**
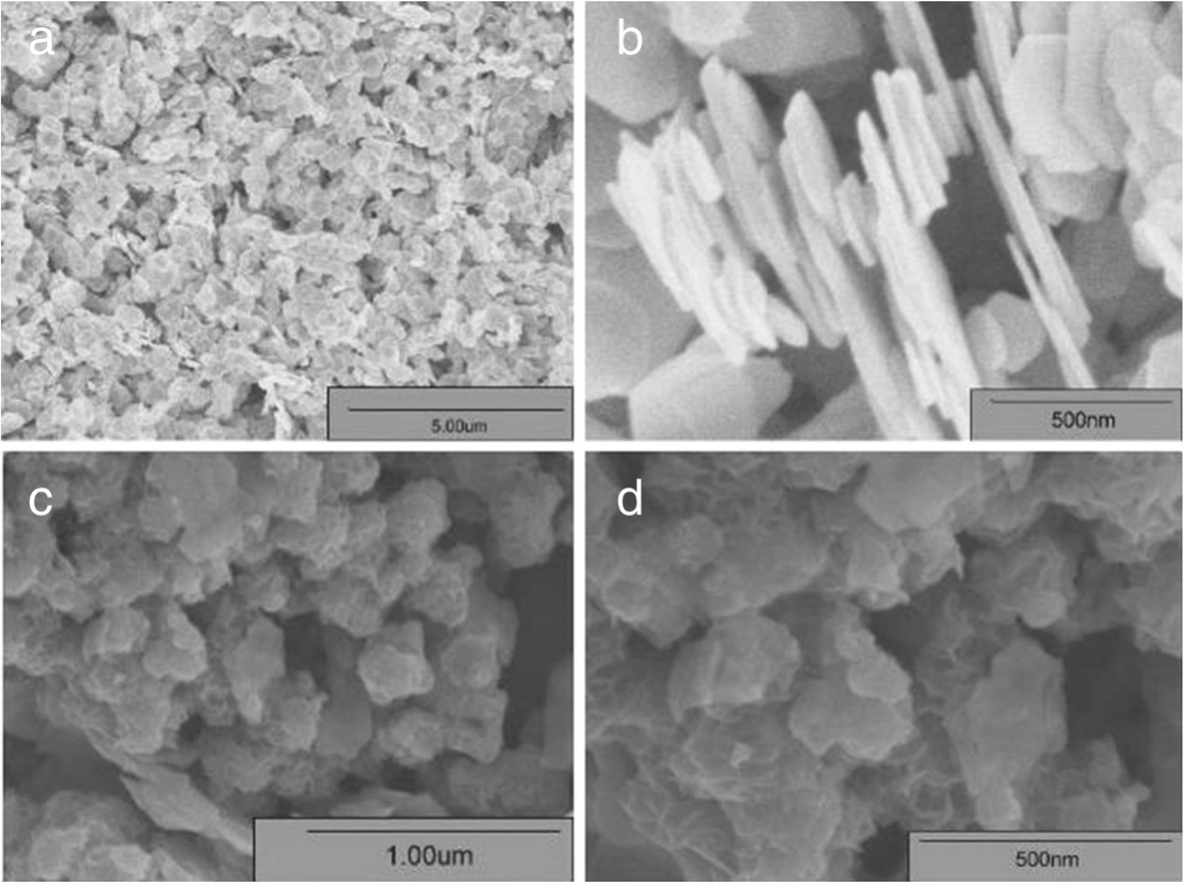
Low-resolution FESEM images of samples (a) A and (c) F and their respective high-resolution SEM images (b, d).

The morphology and size of the as-synthesized products are characterized by FESEM observations (Figure 2). The low-magnification FESEM image in Figure 2a shows that a large number of platelike nanostructures are randomly dispersed on the surface of the substrate. Comparatively, a perfect hexagonal morphology for Bi_2_Se_3_ is observed from the image. A magnified FESEM image (Figure 2b) shows that the width of the nanosheets is in the range of 100 to 400 nm with a thickness of about 10 to 30 nm. The doping of Mo changes the morphologies of the nanosheets greatly. The low-magnified FESEM image (Figure 2c) demonstrates that the typical product of Bi_1.85_Mo_0.15_Se_3_ consists of a large quantity of uniform flowerlike nanospheres. The average diameter of flowerlike nanospheres is about 100 to 200 nm, and they are made up of curved nanoplates with an average thickness of 5 to 10 nm as shown in the high-magnification FESEM image (Figure 2d).

**Figure 3:**
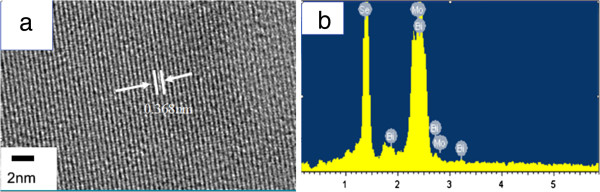
A HRTEM image of sample F (a) and the EDXA spectrum of sample F (b).

To confirm the structure, crystallinity, and details of the flowerlike nanospheres, high-resolution transmission electron microscopy (HRTEM) techniques are employed. A representative HRTEM image taken from the edge of a Bi_1.85_Mo_0.15_Se_3_ nanoflower is shown in Figure 3a, which clearly indicates that nanoflowers contain a perfectly periodic arrangement with an interplanar distance of *a* = 0.356 nm, which is smaller than that of bulk anatase (*a* = 0.413 nm) crystal.

The chemical composition of Bi_2−*x*_Mo_*x*_Se_3_ was determined by energy-dispersive X-ray analysis (EDXA) attached to the FESEM. In Figure 3b, the EDXA spectrum of the Bi_1.85_Mo_0.15_Se_3_ nanosheets shows that the nanosheets contain only Mo, Bi, and Se without any trace of by-products.

**Figure 4:**
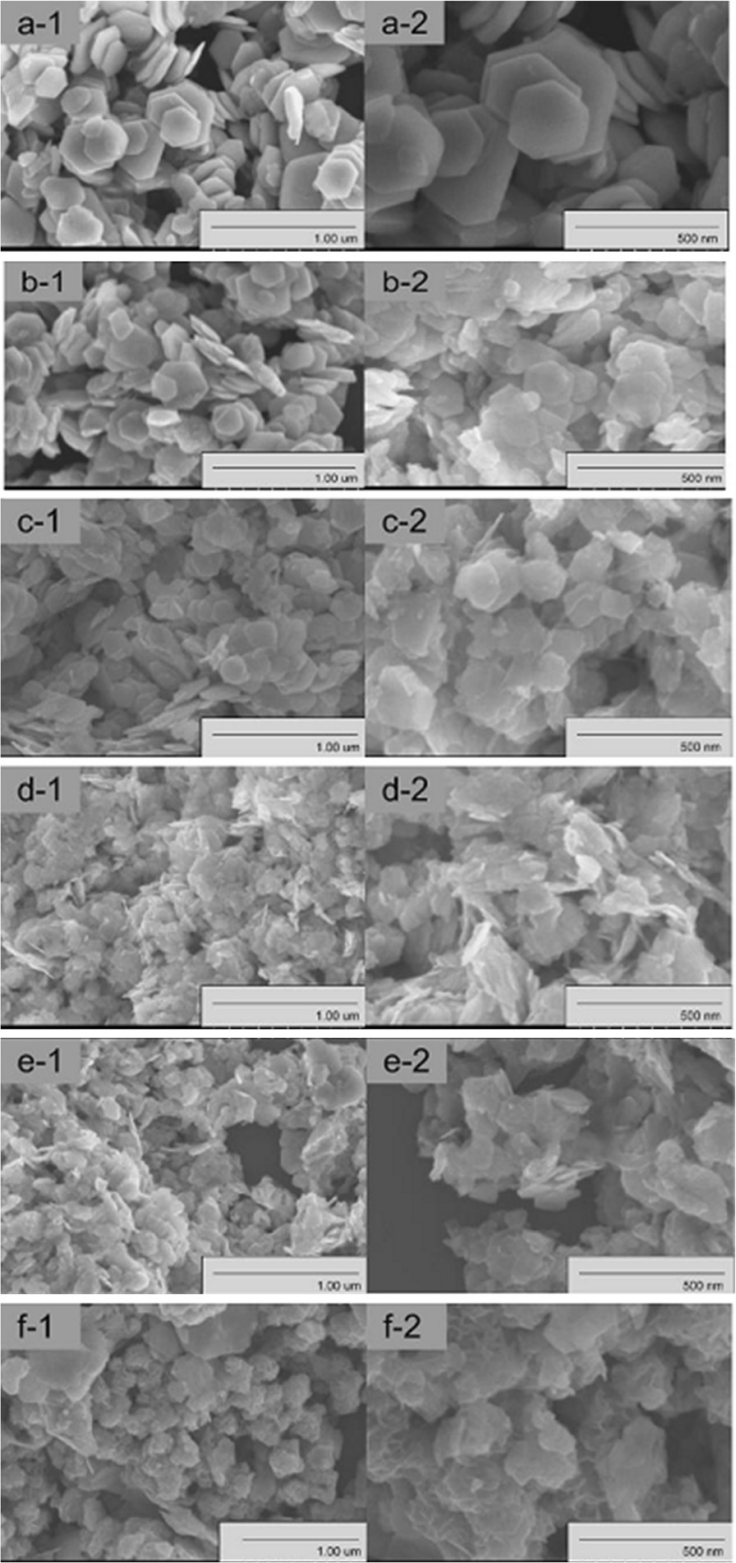
**Low- and high-magnification FESEM images of the samples.** Low-magnification **(a1, b1, c1, d1, e1, and f1)** and high-magnification **(a2, b2, c2, d2, e2, and f2)** FESEM images of samples A **(a1, a2)**, B **(b1, b2)**, C** (c1, c2)**, D **(d1, d2)**, E **(e1, e2)**, and F **(f1, f2)**.

From the FESEM observations, we can conclude that the Mo concentration influences the morphologies of the nanoplates greatly. In order to understand the role of Mo in the evolution process of Bi_2−*x*_Mo_*x*_Se_3_ from nanoplates to nanoflowers, Bi_2−*x*_Mo_*x*_Se_3_ samples with varied *x* values are synthesized and studied. With the increase of the *x* value (Mo concentration), the nanostructures gradually change from hexagonal nanosheets to smaller-sized hexagonal nanosheets and finally to flowerlike spheres, combined with a size change. For example, when no Mo is contained, the sizes of the nanosheet are about 100 to 500 nm in width and 20 to 30 nm in thickness, as shown in Figure 4 (a-1 and a-2). With increasing amounts of Mo, morphologies of the as-synthesized Bi_2−*x*_Mo_*x*_Se_3_ products change from nanosheets to nanoflowers (Figure 4 (a-1 to f-2). From Figure 4 (b-1 to d-2), we can see that the products are still composed of nanosheets, but the sizes of Bi_2_Se_3_ have become smaller. When the Mo concentration increased up to 15% (Figure 4 (f-1 and f-2), regular flowerlike spheres consisting of thin nanoplates were formed. The average diameter of nanoflowers is about 100 to 200 nm, and they are made up of curved nanoplates with an average thickness of 5 to 10 nm as shown in the magnified image. With the increase of Mo contents in Bi_2−*x*_Mo_*x*_Se_3_, the diameter of the products was found to be lower than that of pure Bi_2_Se_3_. So we believe that Mo is the main driving force for the formation of a flowerlike structure.

**Figure 5:**
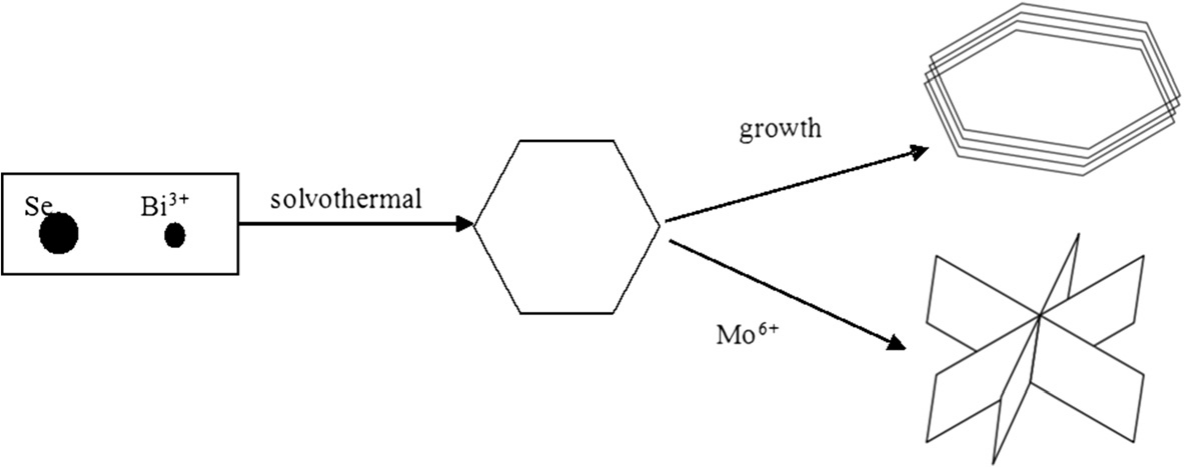
**Schematic diagram showing the growth mechanism of nanosheets and nanoflowers of Bi**_**2−** ***x***_**Mo**_***x***_**Se**_**3**_**.**

A diagram of the formation mechanism of nanoplates and nanoflowers of Bi_2−*x*_Mo_*x*_Se_3_ is presented in Figure 5. When no Mo is contained, tiny clusters of Bi_2_Se_3_ nanosheets are first generated upon heating and then enriched to assemble into bigger nanosheets. However, with the increase of Mo concentration in Bi_2−*x*_Mo_*x*_Se_3_, nanosheets assemble into nanoflowers.

### Adsorption ability of Bi_2−*x*_Mo_*x*_Se_3_

**Figure 6:**
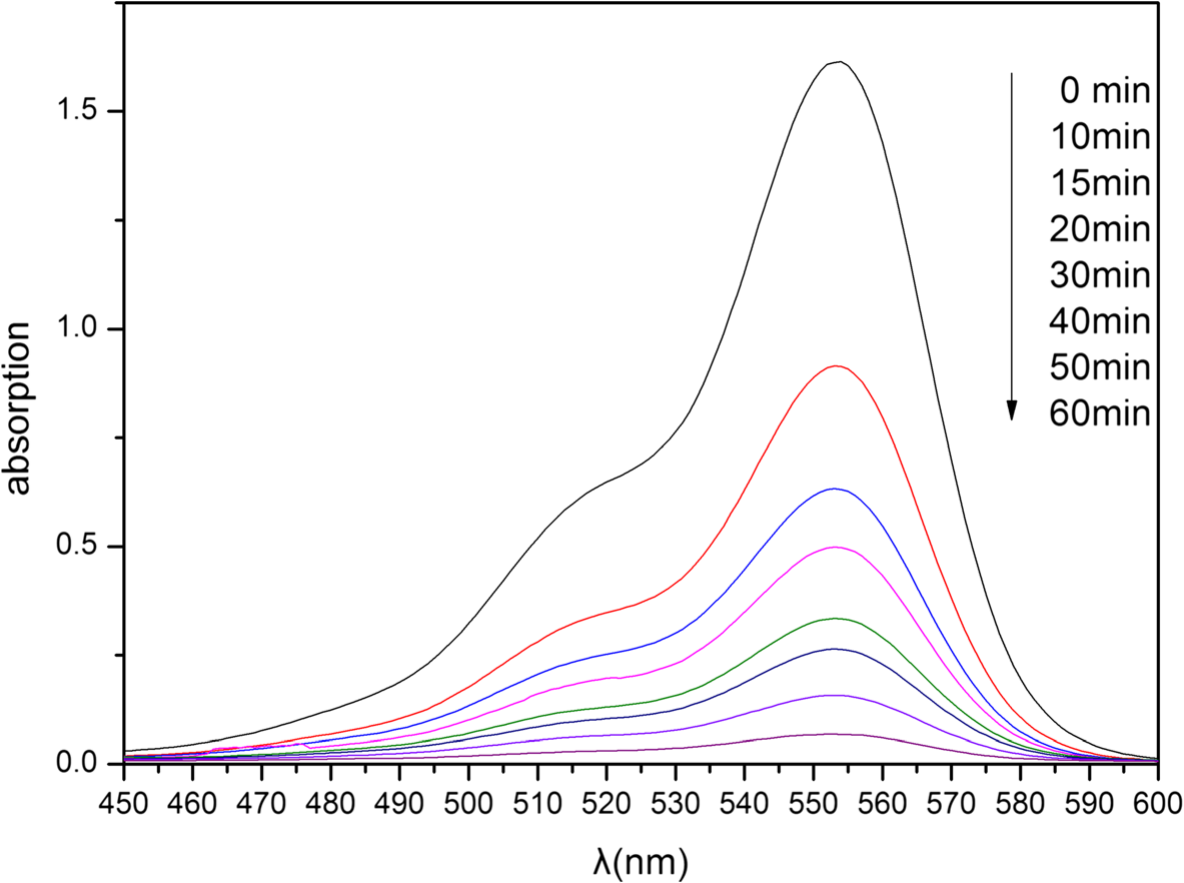
**Time-dependent absorption of 2.0 × 10**^**−4 **^**M 50-ml aqueous solutions of RhB in the presence of 50-mg as-prepared Bi**_**1.99**_**Mo**_**0.01**_**Se**_**3**_**.**

To investigate the potential application of the as-synthesized Bi_2−*x*_Mo_*x*_Se_3_ nanocrystals and their relationship with the amount of Mo in Bi_2−*x*_Mo_*x*_Se_3_, we study the adsorption ability of Bi_2−*x*_Mo_*x*_Se_3_ using RhB as dyes. The experiments are carried out with Bi_2−*x*_Mo_*x*_Se_3_ dispersed in the solution of RhB in the dark several times with constant stirring. After centrifugation, the UV–vis absorption of the supernatant was measured and the characteristic absorption of RhB at about 553 nm was selected to estimate the adsorption process. Figure 6 shows the UV–vis adsorption spectra of RhB as a function of time using the as-prepared Bi_1.99_Mo_0.01_Se_3_ as adsorbent. From Figure 6, we can see that the intensity of the absorption spectra gradually decreases and nearly disappears within 60 min; at the same time, the solution becomes colorless when observed with the naked eye.

**Figure 7:**
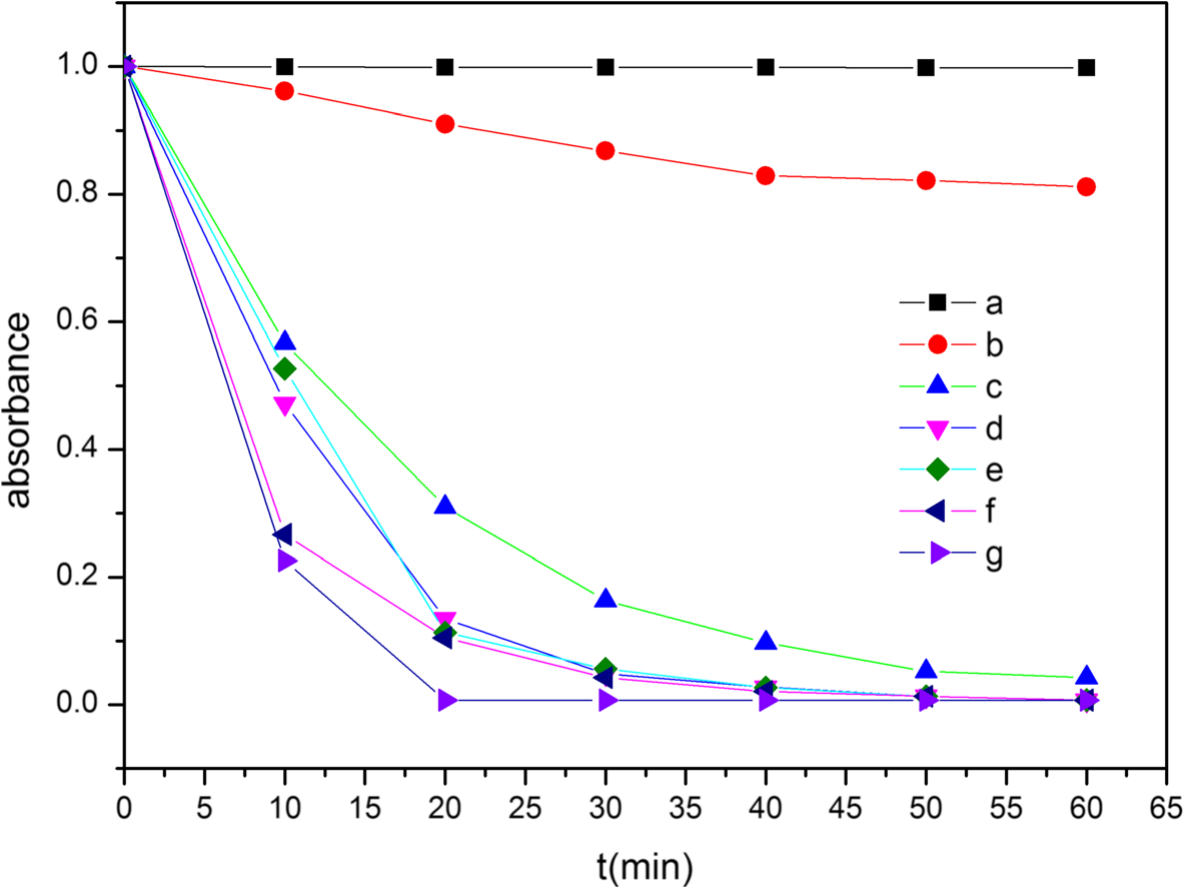
**A plot showing the extent of RhB degradation as a function of adsorption time for different catalysts.** RhB degradation is monitored at 554 nm. Lines **a, b, c, d, e, f,** and **g** represent, respectively, samples blank, A, B, C, D, E, and F.

Figure 7 shows the variation in RhB concentration with the adsorption time over different adsorbents. When there is no adsorbent, the concentration of the RhB solution remains the same with its original state for up to 60 min, which demonstrates that RhB is stable under the experimental conditions. Pure Bi_2_Se_3_ shows a weak adsorption activity. After 60 min of absorption, only 20% of RhB is removed from the pure Bi_2_Se_3_ sample. The adsorption activities are strengthened with the increase of Mo contents in Bi_2_Se_3_. Bi_1.85_Mo_0.15_Se_3_ has a maximum adsorption behavior, and nearly 100% of the RhB dyes are removed in 20 min. All of this clearly shows that the doping of Mo in Bi_2_Se_3_ is an efficient way to enhance its adsorption activity. The results indicate that the as-synthesized Bi_2−*x*_Mo_*x*_Se_3_ might possess a profound application in the fields of treatment of dye-polluted wastewater.

## Conclusions

In summary, Bi_2−*x*_Mo_*x*_Se_3_ nanomaterials were prepared by a solvothermal approach, and different morphologies of Bi_2−*x*_Mo_*x*_Se_3_ have been obtained. The doping concentration of Mo plays an important role in controlling both the morphologies of Bi_2−*x*_Mo_*x*_Se_3_ nanostructures and their absorption behavior. The sample with the best absorption behavior is that with 15% Mo concentration. We believe that the study of dye absorption behavior brings a new application realm for Bi_2_Se_3_ nanostructures.

## Competing interests

The authors declare that they have no competing interests.

## Authors’ contributions

MZ, XM, and JL designed the experiments. MZ and XM performed the experiments. MZ, FW, and YF analyzed the data. MZ made the figures. MZ and XM wrote the manuscript. All authors read and approved the final manuscript.
